# A Case of Methotrexate Neurotoxicity Presented as Status Epilepticus, Encephalopathy, and High Fever

**DOI:** 10.1177/2324709619862311

**Published:** 2019-07-18

**Authors:** Itay Ayalon, Shirley Friedman, Yoav Binenbaum, Noga Oppenheimer, Shelly Shiran, Galia Grisaru-Soen, Shimrit Uliel-Sibony, Miguel Glatstein, Jennifer Melissa Kaplan, Efraim Sadot

**Affiliations:** 1 “Dana-Dwek” Children’s Hospital, Tel Aviv, Israel; 2 Cincinnati Children’s Hospital Medical Center, Cincinnati, OH, USA

**Keywords:** aminophylline, fever, methotrexate, neurotoxicity, status epilepticus

## Abstract

High-dose methotrexate is used to treat a range of adult and childhood cancers including osteosarcoma. Significant neurotoxicity is reported in 1% to 4.5% of patients treated with high-dose methotrexate and can present in a wide variety of symptoms. We present a case of a 14-year-old boy with a recent diagnosis of osteosarcoma who presented to the emergency department with status epilepticus, altered mental status, and very high fever secondary to methotrexate neurotoxicity. We review current literature and discuss some controversies related to this state. We also describe high fever as one of the possible symptoms associated with this condition and suggest using specific magnetic resonance imaging sequence to uncover abnormal findings related to this state. Since high-dose methotrexate is not a rare treatment in this era, we believe that in addition to oncologists, emergency department and intensive care providers should be aware of the potential role of methotrexate in causing significant neurotoxicity and include it in the differential diagnosis when treating a patient presenting with new neurological symptoms in the setting of recent high-dose methotrexate treatment.

## Introduction

Status epilepticus (SE) and altered mental status (AMS) are among the most common emergency states encountered by emergency department (ED) and intensive care providers. These states often require prompt evaluation and aggressive stabilization done in a timely manner and per the patient’s background and circumstances surrounding his/her presentation. In some cases, the etiology of SE and AMS is obvious (eg, head trauma, substance abuse, and sepsis) while in other cases the etiology is more obscure, necessitating more thorough “digging” into the patient history and more use of ancillary tests.

In this report, we present a case of a 14-year-old boy with a recent diagnosis of osteosarcoma who presented to the ED with SE and AMS secondary to subacute methotrexate (MTX) neurotoxicity 5 days after he finished his second dose of intravenous high-dose MTX (HDMTX) treatment. We review current literature and discuss some controversies related to this state. We also describe high fever as one of the possible symptoms associated with this condition and suggest using specific magnetic resonance imaging (MRI) sequence to uncover abnormal findings related to MTX neurotoxicity.

## Case Presentation

A 14-year-old boy with a recent diagnosis of osteosarcoma of the right tibia without any known metastasis presented to the ED with SE and AMS. Five days prior to his presentation the patient underwent his second course of HDMTX (12 000 mg/m^2^, single dose, based on the American Osteosarcoma Study Group 0331 [EURAMOS-1] protocol [ClinicalTrials.gov Identifier: NCT00134030]). Twenty-four hours following HDMTX he had a generalized tonic-clonic seizure for the first time in his life. The seizure lasted 5 minutes and was stopped pharmacologically with a single dose of midazolam. His electroencephalography was normal. MTX levels were followed and found to be in the nontoxic range (1.7, 0.13, 0.06 µmol/L at 24, 48, and 72 hours following injection, respectively). Seizures did not recur for 48 hours and the patient was discharged home on levetiracetam treatment.

**Figure 1.**
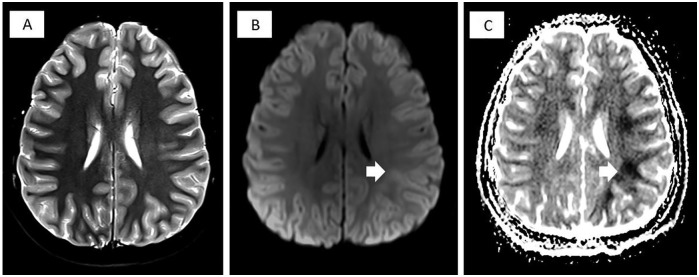
Brain magnetic resonance imaging of a patient with methotrexate neurotoxicity showing very subtle signal changes on the T2-weighted imaging (A), mild increased signal on diffusion weighted imaging (DWI; B, white arrow), and prominent decreased signal on apparent diffusion coefficient (ADC) map on the left posterior subcortical white matter area (C, white arrow).

One day after his discharge home, the patient had once again generalized tonic-clonic seizure. Emergency medical services were called and found the patient seizing. He was treated with 3 doses of midazolam. On arrival to the ED he was still seizing and was treated with a single dose of diazepam and a loading dose of levetiracetam after which his seizure stopped. On initial examination, the patient was obtunded, responding only to painful stimuli with symmetric limb movement (localizing pain), eye opening, and moaning. His initial glucose and electrolytes, kidney, and liver function tests were all normal. Arterial blood gas revealed respiratory acidosis (pH = 7.15, pCO_2_ = 93 mm Hg, pO_2_ = 150 mm Hg [with oxygen supplementation], base excess = 2.7) that resolved completely as the patient stopped seizing. His initial complete blood count showed leukocytosis and elevated neutrophils (white blood count = 21 600 cells/µL, 90% neutrophils) and C-reactive protein was mildly elevated (12 mg/L [normal = 0-5 mg/L]). Head computed tomography was normal without any focal findings. A subsequent brain MRI revealed subtle diffusion restriction in the posterior subcortical white matter, more prominent on the left periventricular white matter, extending to the parietotemporal and centrum semiovale areas. Signal changes were more prominent on the apparent diffusion coefficient map than on the diffusion-weighted imaging (Figure 1). No evidence of territorial infarct or metastases was found. During his stay in the ED the patient had high temperatures reaching 39.5°C (103.1°F). Cerebrospinal fluid obtained via a lumbar puncture demonstrated marginally elevated white count (10 white cells/µL, 75% polymorphonuclear neutrophils) with normal glucose (74 mg/dL) and protein (38 mg/dL). The patient was started on broad spectrum antibiotics (piperacillin/tazobactam and amikacin) in combination with acyclovir. Blood and cerebrospinal fluid were sent for bacterial cultures and herpes simplex virus, varicella zoster, and enterovirus polymerase chain reactions studies. All polymerase chain reactions and cultures were found negative.

The patient was admitted to the pediatric intensive care unit for close neurologic monitoring with a probable diagnosis of MTX neurotoxicity. He received 4 doses of aminophylline (2.5 mg/kg/dose per day for 4 consecutive days) in combination with high-dose steroids (dexamethasone). During his stay in the pediatric intensive care unit the patient remained seizure free, did not require hemodynamic or respiratory support, and continued to have persistent high fevers. Due to mild skin erythema and limb swelling near his central venous catheter, the catheter was removed and the patient completed 7 days of intravenous antibiotics for a “rule out” cellulitis. Repeated electroencephalography while obtunded, showed paroxysmal generalized delta wave activity with epileptiform activity in the temporal lobe consistent with encephalopathy. There was no improvement of the neurological examination until the fourth day of admission (~72 hours following presentation to the ED) when the patient was able to open his eyes spontaneously and follow verbal commands. At that time his neurologic examination was significant for central facial palsy, relative weakness of his left upper and lower extremities (motor strength of 3-4/5), and anisocoria of his pupils (left > right). The patient underwent a repeated head computed tomography with angiography that did not show any signs for vascular stroke or diminished perfusion. The new neurological findings were attributed to MTX neurotoxicity versus Todd’s paresis. In the next few days, the patient recovered almost completely with only minimal residual neurological deficits (anisocoria, facial asymmetry, and instability on tandem gait). His fever subsided by day 4 and he was transferred to the oncology floor for further observation.

## Discussion

High-dose MTX, defined as a dose of 500 mg/m^2^ or higher, is used to treat a range of adult and childhood cancers including acute lymphoblastic leukemia, central nervous system lymphomas, leptomeningeal metastases, and osteosarcoma.^1-4^

MTX acts as an antimetabolite by interfering with the metabolism of folic acid. It binds to the intracellular enzyme dihydrofolate reductase with an affinity that is about 1000-fold greater than folate. By doing so, MTX indirectly inhibits the conversion of dihydrofolate to tetrahydrofolate (THF), and since THF has an essential role in DNA synthesis, blockade of THF synthesis leads to inability of cells to produce proteins and divide.^5^ Of note, neoplastic cells often have innate or acquire resistance to MTX, which may hamper MTX efficacy and can lead to treatment failure. At least 5 mechanisms had been suggested to explain this phenomenon, one of which is decreased MTX accumulation inside the cells due to impaired transporters function.^6^ In addition, several MTX transporters polymorphisms had been linked to higher plasma levels of MTX and worse prognosis.^7^

HDMTX therapy can cause significant toxicity, which can lead to substantial morbidity and mortality. Nephrotoxicity, hepatotoxicity, pulmonary toxicity, dermatologic toxicity, and neurotoxicity have all been described in association with MTX treatment.^8-12^ With regard to HDMTX neurotoxicity, neurological symptoms are reported in 1% to 4.5% of patients receiving HDMTX within 2 weeks of initiation of treatment. Symptoms can follow an acute, subacute, or chronic course with variable manifestations including hemiparesis or other stoke-like symptoms, ataxia, dysphasia, encephalopathy, seizures, headaches, and weakness.^13,14^ In cases of acute or subacute encephalopathy, the most frequently seen neurotoxicity following HDMTX, clinical symptoms are often associated with leukoencephalopathy (white matter hyperintensity on T2-weighted and fluid attenuated inversion recovery MRI).^13,15^ Interestingly, leukoencephalopathy can be found in as many as 20% of asymptomatic patients treated with HDMTX.^14^ The pathogenesis of HDMTX-associated neurotoxicity is not clear, although several hypotheses have been proposed, including homocysteine toxicity, altered folate homeostasis, adenosine release, and/or direct neuronal damage by MTX.^16-18^

Currently there is no standard treatment for neurotoxicity related to HDMTX. Small series suggest neurological symptoms can be relieved by aminophylline (2.5 mg/kg) via competitive inhibition of adenosine^18^ and dextromethorphan (1-3 mg/kg), an N-methyl-D-asparate receptor antagonist that inhibits homocysteine activity.^19,20^ The role of steroids in this setting is not clear. HDMTX neurotoxicity with low blood levels of MTX should be differentiated from neurotoxicity associated with MTX overdose with toxic blood levels of MTX. In MTX overdose intrathecal administration of carboxypeptidase G2 (CPDG2) is warranted. CPDG2 rapidly hydrolyzes MTX to inactive metabolites.^21^ Other treatments that had been suggested in cases of overdose are cerebrospinal fluid drainage, ventriculolumbar perfusion, systemic leucovorin administration, and urine alkalization.^21,22^ There is no evidence that CPDG2 and the other mentioned modalities have a role in neurotoxicity in the absence of toxic blood levels of MTX.

Acute and subacute encephalopathy related to HDMTX is usually transient, with recovery occurring within 1 to 7 days of symptoms onset. Chronic encephalopathy develops more slowly and may result in permanent neurological deficits. Whether to rechallenge patients with MTX related neurotoxicity with repeated doses of HDMTX is a matter of debate. Bhojwani et al^14^ followed 369 children with ALL who were treated with HDMTX of which 14 exhibited MTX-related neurotoxicities. Most episodes were brief, and all but one patient were successfully rechallenged with high-dose MTX and/or intrathecal administration of MTX after resolution of symptoms. The authors concluded that stopping MTX treatments following episodes of resolved neurotoxicity is unnecessary.^14^

In our case report, we describe a patient who presented to the ED with SE and AMS/encephalopathy who underwent an extensive evaluation leading to the diagnosis of MTX neurotoxicity. Other important etiologies like brain metastases, brain hemorrhage, ischemic stroke, sepsis, and electrolytes abnormalities were excluded. For the most part his clinical course followed the known course of acute/subacute neurotoxicity related to MTX as described above. The only exception was his consistent high fevers in the absence of clear source of infection (the redness and swelling of his left arm were only mild and not impressive enough to explain the high fevers he had) that resolved in parallel to the improvement in his neurological examination. As far as we know this is the first report associating high fever with MTX neurotoxicity. We also suggest that using apparent diffusion coefficient sequence on MRI can potentially uncover abnormal signals that are not clearly and easily seen on other sequences. Of note, possible polymorphisms of MTX transporters were not tested in this case.

HDMTX is not a rare treatment in this era. In addition to oncologists, ED and ICU providers should be aware of its potential role in causing significant neurotoxicity and include it in the differential diagnosis when treating a patient presenting with new neurological symptoms in the setting of recent HDMTX treatment.
